# Supplementary material for: Integrated Analysis of the Lung Microbiome and Metabolome Reveals Associations Between Amino Acid Metabolism and Pulmonary Fibrosis in a Bleomycin-Induced Mouse Model
Source: Int J Mol Sci. 2026 Jun 30;27(13):5895. doi: 10.3390/ijms27135895 (PMC13362081; doi:10.3390/ijms27135895)
Supplement: Supplementary file 1 [file ijms-27-05895-s001.zip › result/5.MetKeggEnrichment/B_14d.vs.C_14d/B_14d.vs.C_14d_all_KEGG_classification.pdf]

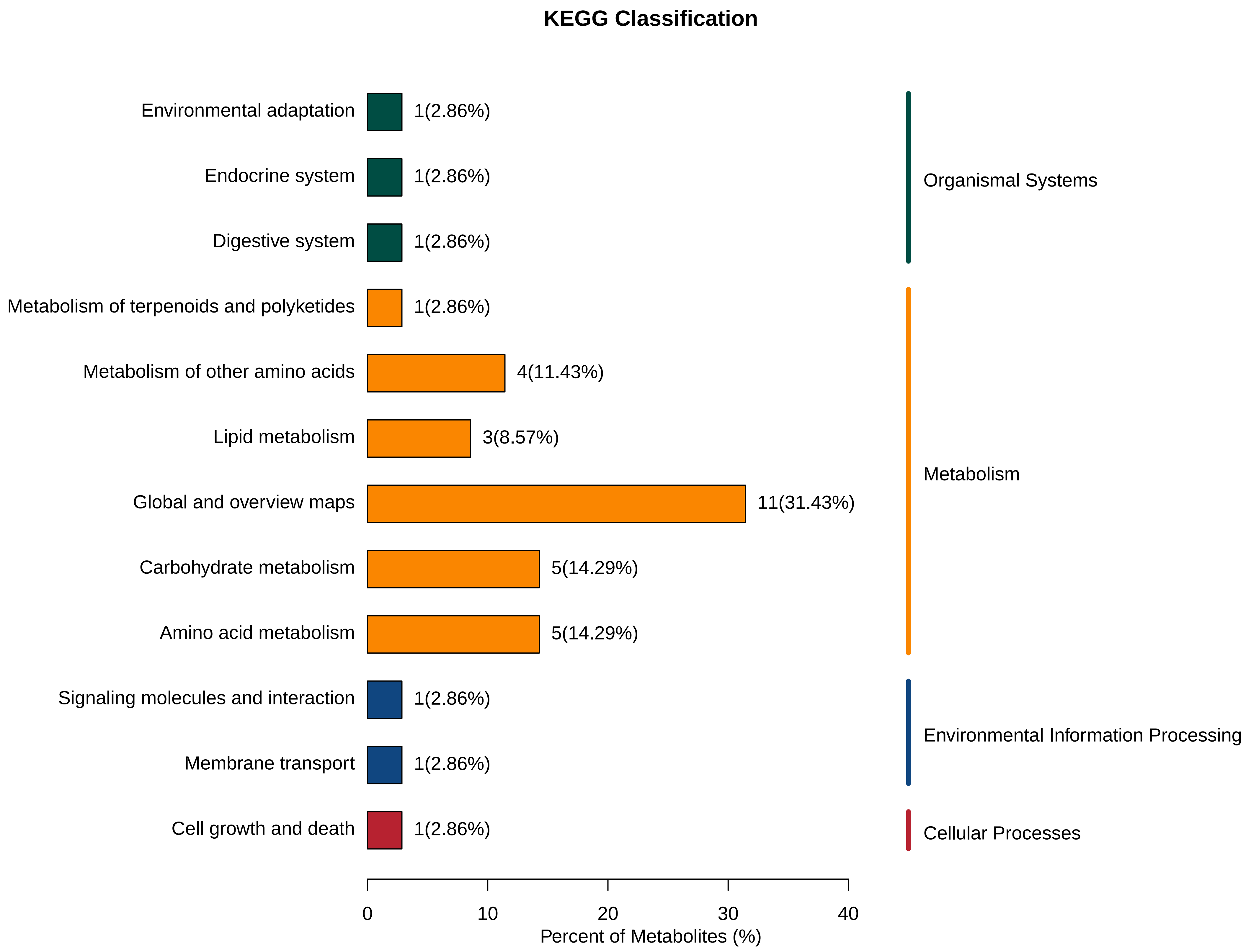
KEGG Classification
Environmental adaptation
1(2.86%)
Endocrine system
1(2.86%)
Digestive system
1(2.86%)
Metabolism of terpenoids and polyketides
1(2.86%)
Metabolism of other amino acids
4(11.43%)
Lipid metabolism
3(8.57%)
Global and overview maps
11(31.43%)
Carbohydrate metabolism
5(14.29%)
Amino acid metabolism
5(14.29%)
Signaling molecules and interaction
1(2.86%)
Membrane transport
1(2.86%)
Cell growth and death
1(2.86%)
Organismal Systems
Metabolism
Environmental Information Processing
Cellular Processes
0
10
20
30
40
Percent of Metabolites (%)
